# Supplementary material for: Metabolomic Strategy to Characterize the Profile of Secondary Metabolites in Aspergillus aculeatus DL1011 Regulated by Chemical Epigenetic Agents
Source: Molecules. 2022 Dec 26;28(1):218. doi: 10.3390/molecules28010218 (PMC9821969; doi:10.3390/molecules28010218)
Supplement: Supplementary file 1 [file molecules-28-00218-s001.zip › molecules-2023025-supplementary.pdf]

Metabolomic strategy to characterize the profile of secondary metabolites in *Aspergillus aculeatus* DL1011 regulated by chemical epigenetic agents

supplementary material

Xuan Shi [1], Yu Sun [1], Junhui Liu [1], Wencai Liu [2], Yan Xing [1], Zhilong Xiu [1] and Yuesheng Dong [1*]

1 School of Bioengineering, Dalian University of Technology, Dalian 116024, China; xuan80904@mail.dlut.edu.cn (X.S.); yu.sun@bbctg.com.cn (Y.S.); junhui.liu@pharmaron-bj.com (J.L.); docxyxy@163.com (Y.X.); zhlxiu@dlut.edu.cn (Z.X.)
2 Shandong Provincial Engineering Laboratory of Protein Pharmaceutical, Shandong New Time Pharmaceutical Co. Ltd., Linyi, 273400, China; liuwencai1986@163.com
* Correspondence: yshdong@dlut.edu.cn.

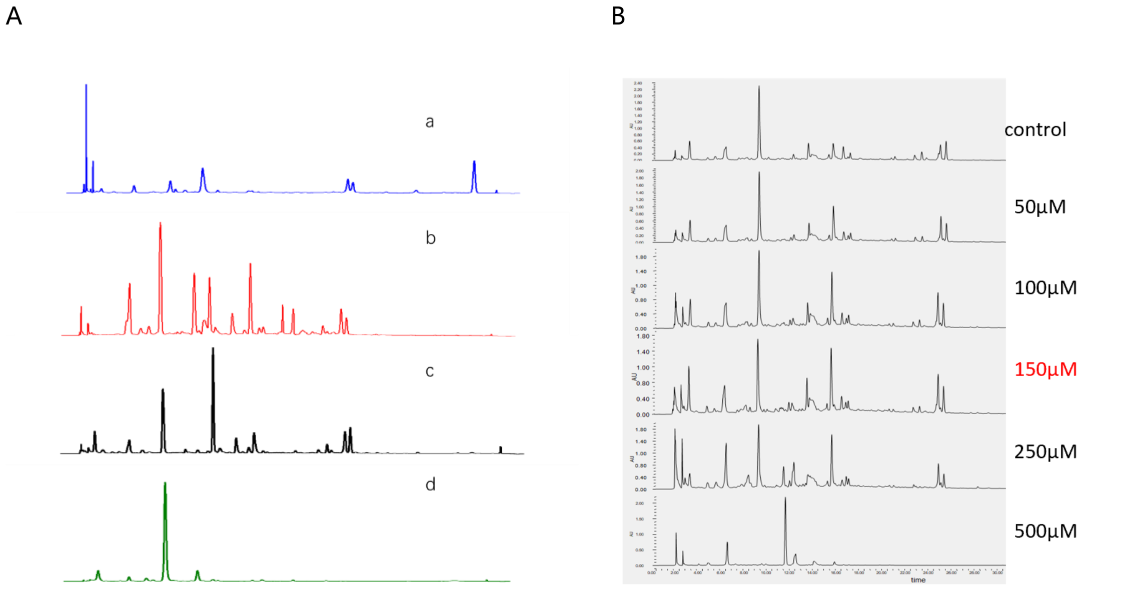

Fig. S1A. HPLC profile of extracts of *Aspergillus aculeatus* DL1011 cultivated with 150 μM (b)SBHA, (c)SAHA, (d) nicotinamide and (a) control detected by UV absorption at 260 nm; B. HPLC profile with different concentrations of SBHA treatment.

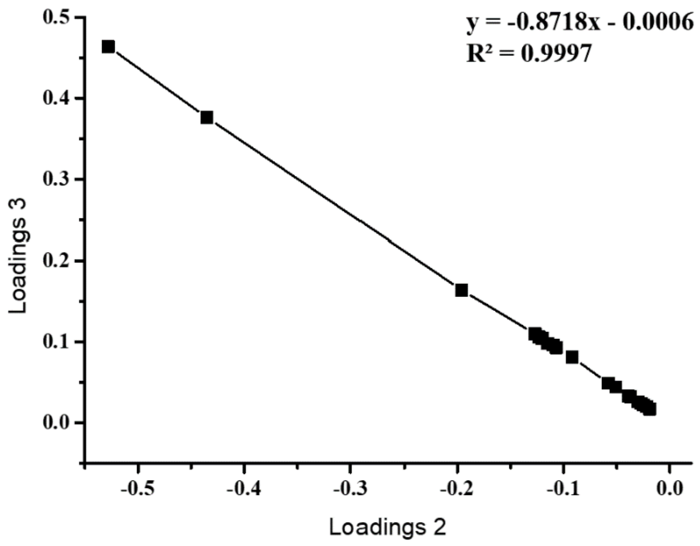

Fig. S2 Features produced by CER shown linear correlation.

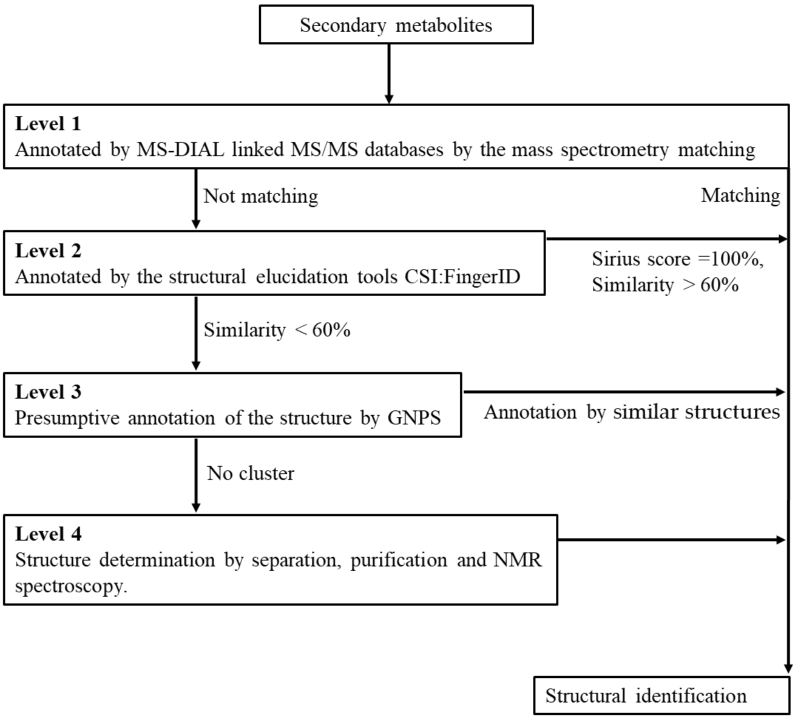

Fig.S3 Process of metabolites identification.

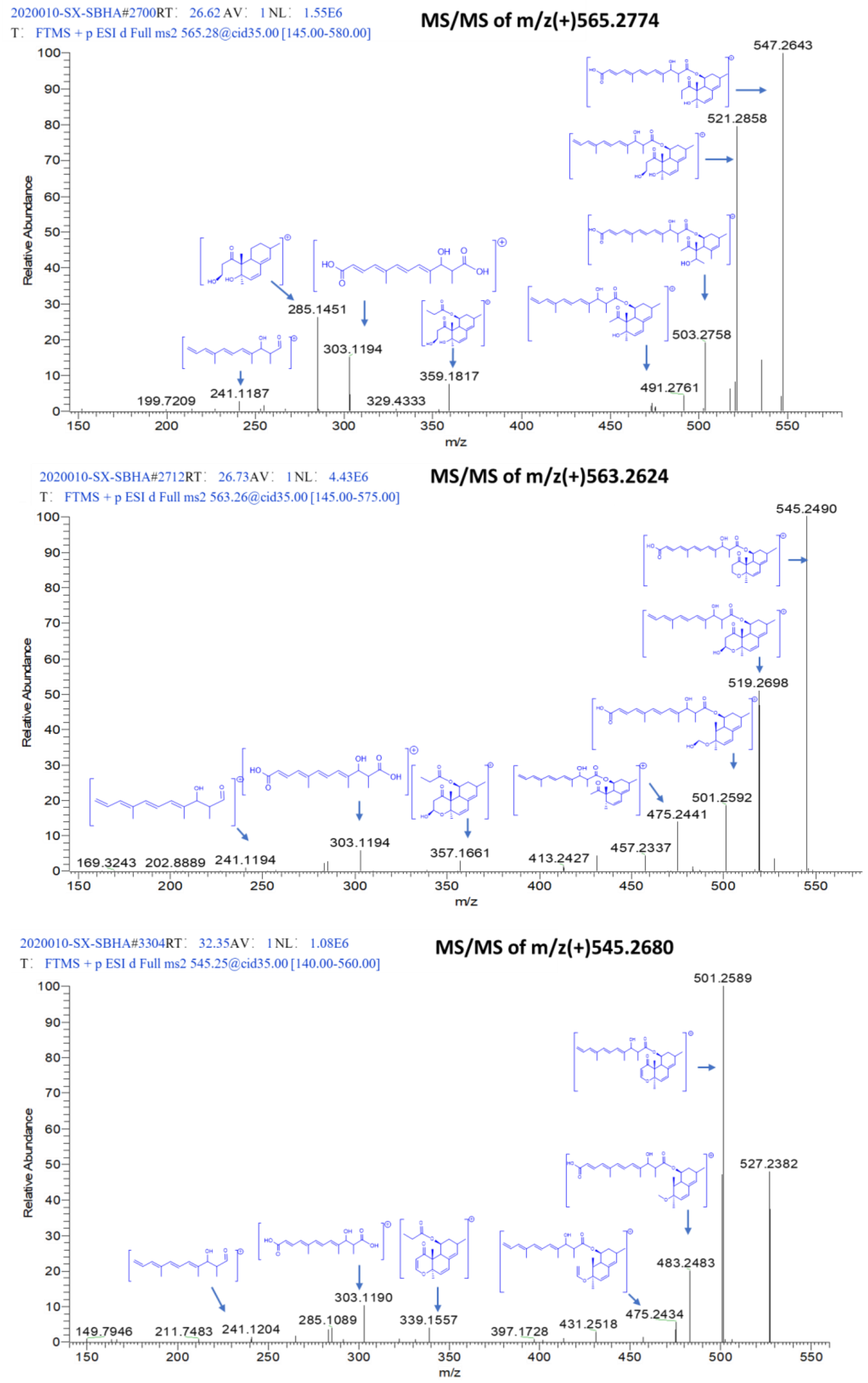

Fig.S4 Annotated MS/MS spectrum of m/z 545.2680 (**S16**), 563.2624 (**S17**), and 565.2774 (**S18**) acquired by LTQ-Orbitrap-XL in positive mode.

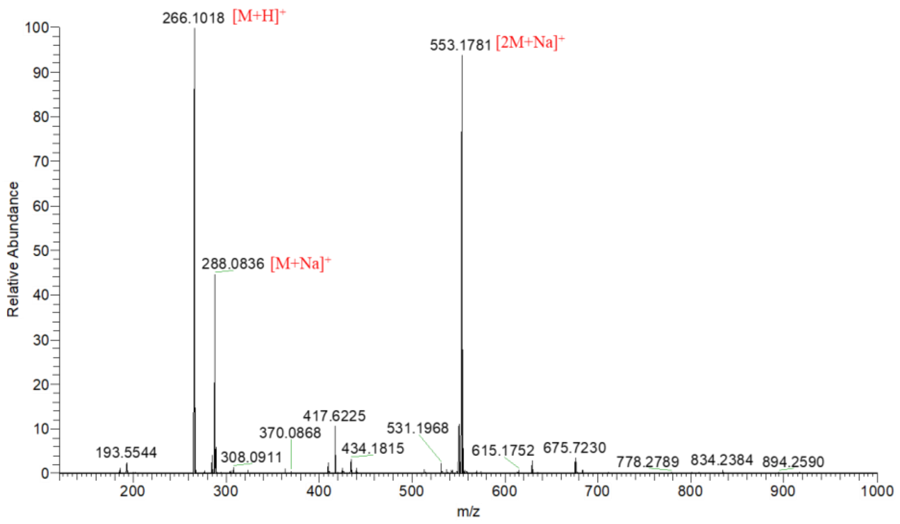

Fig.S5 Positive HRESIMS spectrum of **S5**

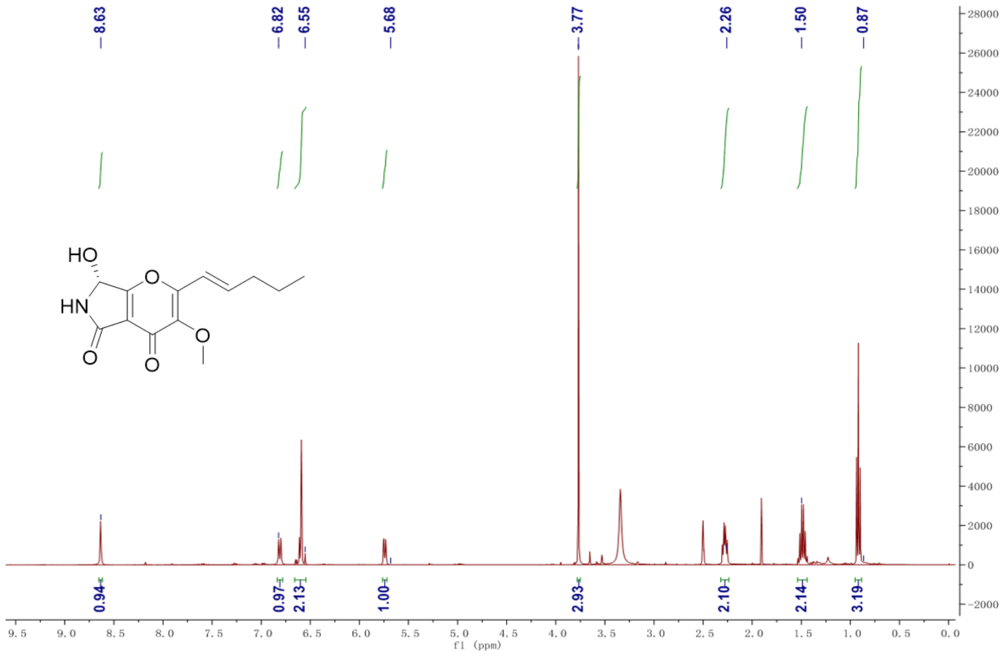

Fig.S6 ^{1}H NMR (500 MHz, DMSO-d_6) spectrum of pyranonigrin G (**S5**).

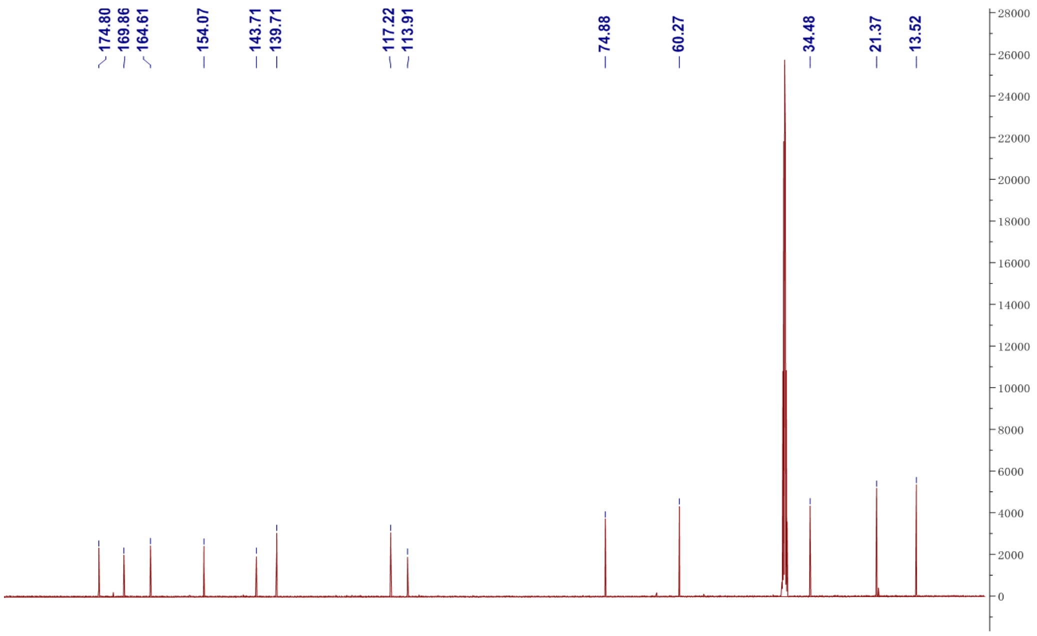

Fig.S7 ^{13}C NMR (250 MHz, DMSO-d_6) spectra of pyranonigrin G(**S5**).

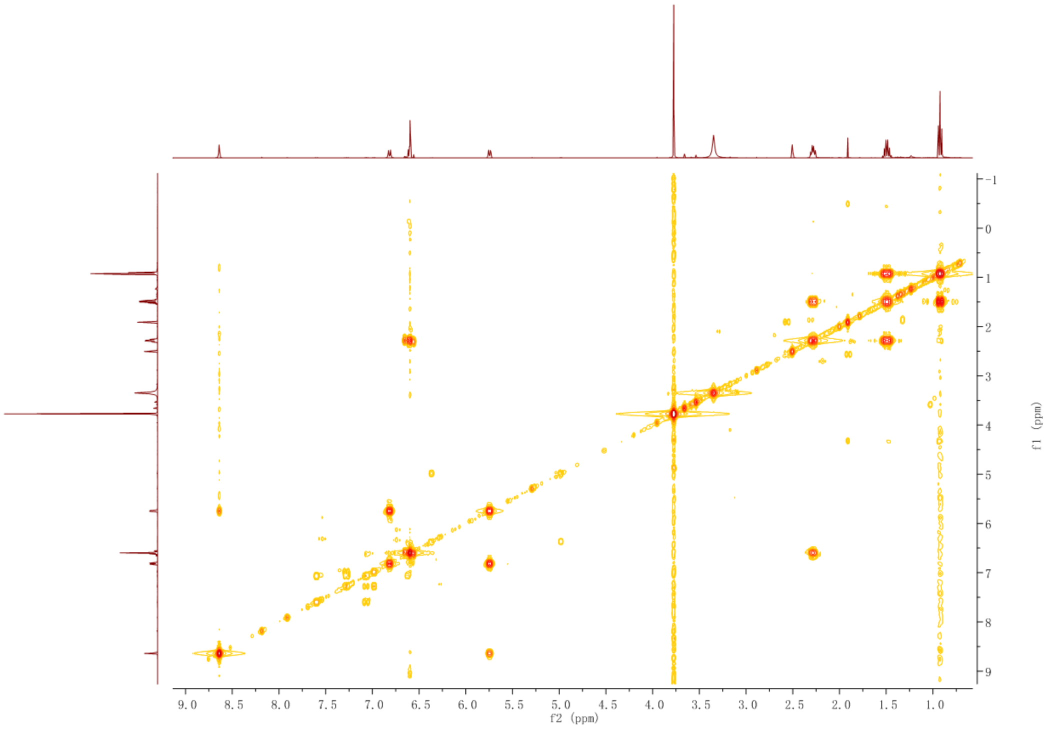

Fig.S8 H-H COSY spectrum of pyranonigrin G (**S5**).

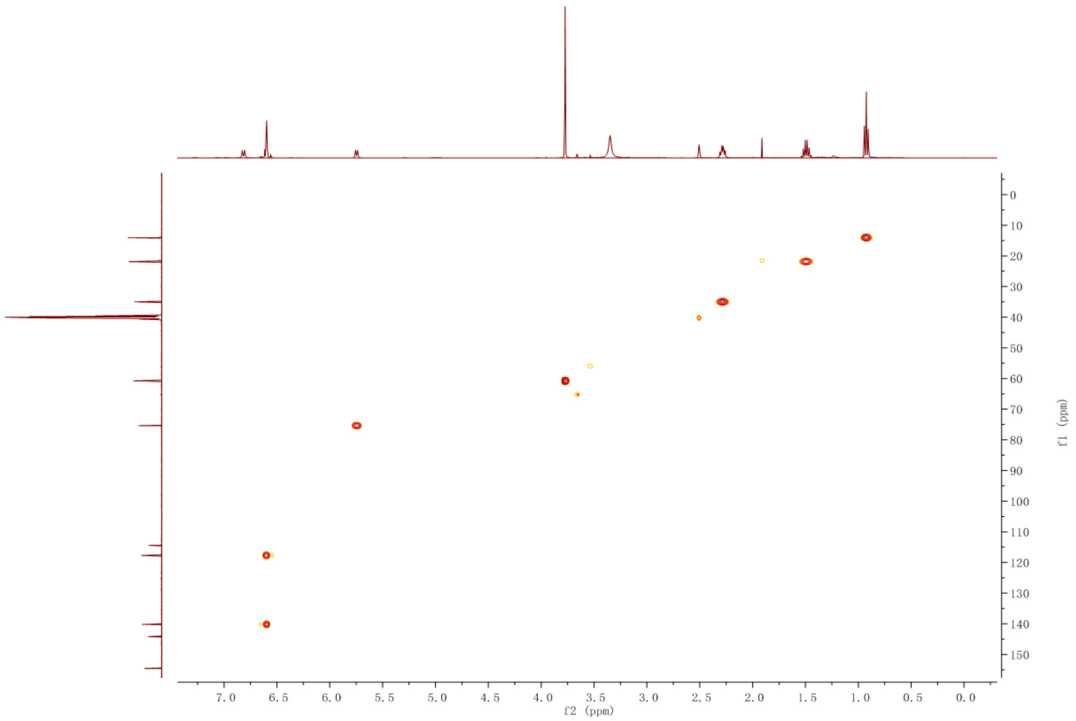

Fig.S9 HMQC spectrum of pyranonigrin G (**S5**).

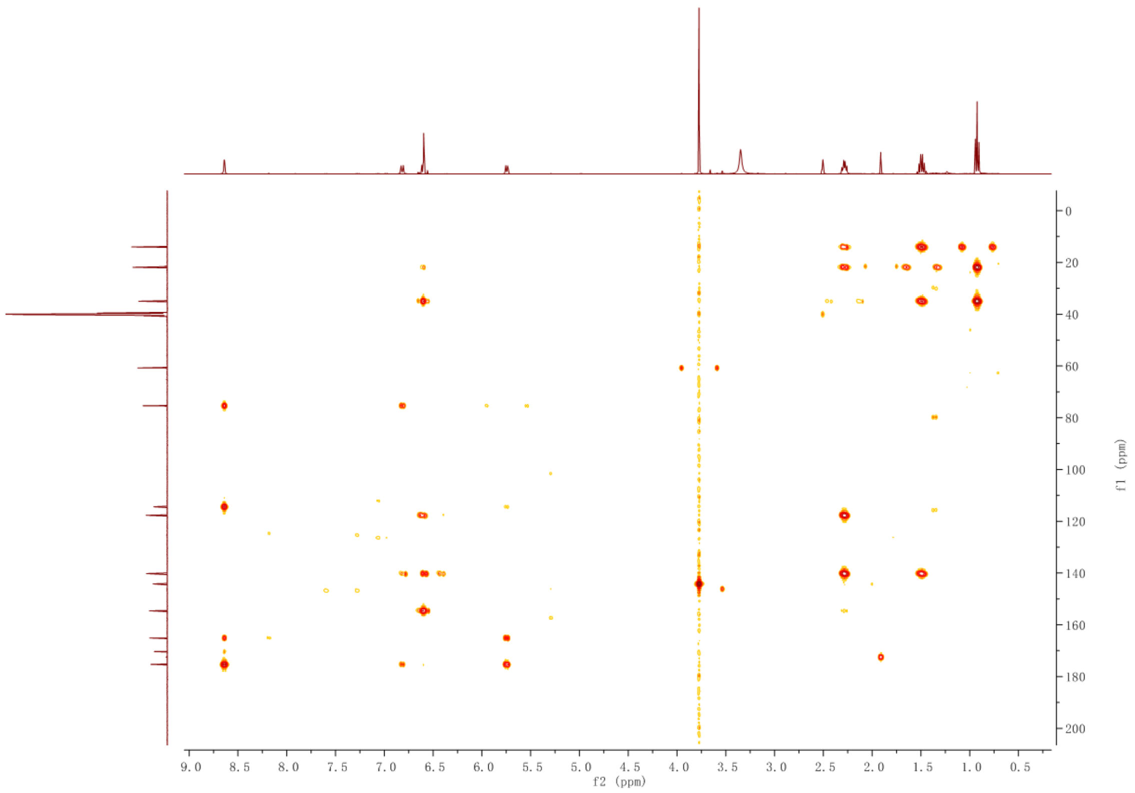

Fig.S10 HMBC spectrum of pyranonigrin G (**S5**).

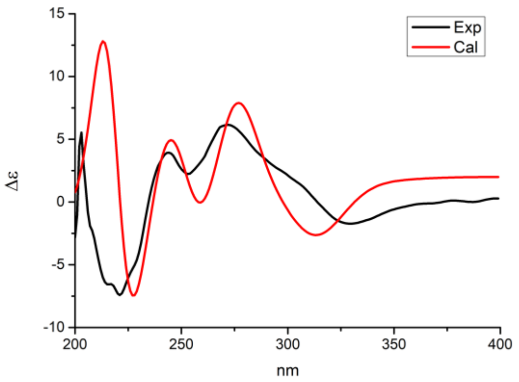

Fig. S11 Calculated and experimental ECD spectra of pyranonigrin G (**S5**) (red, calculated at the B3LYP/6-31G(d)//B3LYP/6-31G (d, p) level in MeOH; black, experimental in MeOH).

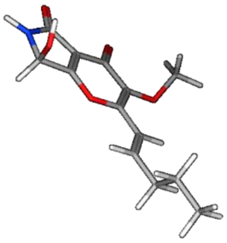

S5-1

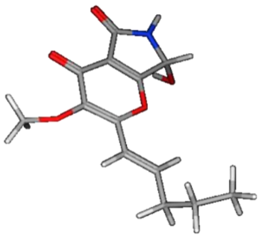

S5-2

Fig.S12 Optimized conformers (≥ 1%) of pyranonigrin G (**S5**) at the B3LYP/6-31+G (d) level with PCM model in MeOH.

Table S1. Important Thermodynamic Parameters and Conformational Analysis of pyranonigrin G (**S5**).

Conformations	E+ZPE	G	P
S5-1	-934.969575	-934.988547	88.40%
S5-2	-934.969575	-934.969575	11.60%

E+ZPE: total energy with zero point energy; G: Gibbs free energy; P: conformational distributions calculated from relative Gibbs free energy.

Table S2. Optimized Cartesian Coordinate of compound pyranonigrin G (**S5**) at B3LYP/6-31G (d, p) level in MeOH Using the PCM Model

No.	Atom	S5-1			S5-2		
		X	Y	Z	X	Y	Z
1	C	-1.88181	-2.38865	-0.18697	1.890525	-2.30575	0.274552
2	C	-1.30939	-0.98421	-0.21162	1.244396	-0.948907	0.069961

3	C	6.510192	-0.81487	0.364712	-5.014159	-0.489682	1.602077
4	C	-2.25512	-0.02671	-0.09263	2.130172	0.070354	0.11517
5	C	4.989334	-0.92077	0.510986	-5.102909	-1.237645	0.26861
6	C	4.235407	-0.21458	-0.63553	-4.280015	-0.585946	-0.865125
7	C	-0.11848	3.667619	0.890815	-0.23873	3.73327	0.425405
8	C	2.748106	-0.36475	-0.52971	-2.800879	-0.624472	-0.622451
9	C	-1.89932	1.386625	-0.09569	1.706228	1.445542	-0.116818
10	C	0.448231	0.526515	-0.29208	-0.573606	0.427567	-0.351306
11	C	-0.43643	1.573467	-0.19294	0.246332	1.530293	-0.332051
12	C	-3.57725	-0.71976	-0.00993	3.475305	-0.525747	0.382686
13	C	1.88666	0.661449	-0.40805	-2.004138	0.459007	-0.582342
14	H	2.364527	-1.38423	-0.542	-2.365068	-1.611527	-0.470601
15	H	7.022205	-1.31998	1.191365	-5.624791	-0.97985	2.368652
16	H	6.85011	-1.27417	-0.57152	-3.982664	-0.448149	1.970436
17	H	-4.01112	-2.76045	-0.14482	3.986356	-2.517593	0.760159
18	H	-1.50398	-2.83639	1.662364	1.996926	-2.874759	-1.57721
19	H	4.67515	-0.48397	1.467746	-6.149804	-1.289055	-0.055919
20	H	-1.71367	-2.90321	-1.13913	1.532042	-2.783403	1.192627
21	H	2.245328	1.685514	-0.39563	-2.413892	1.452455	-0.732891
22	H	0.383632	3.224029	1.758133	-0.759886	3.383224	1.323689
23	H	4.692869	-1.97791	0.540202	-4.769912	-2.275994	0.40189
24	H	6.835579	0.23253	0.359268	-5.371149	0.54265	1.499342
25	H	-1.18084	3.806318	1.098106	-0.743928	4.615717	0.029656
26	H	0.346079	4.624846	0.648864	0.801565	3.966629	0.657892
27	H	4.570061	-0.65219	-1.5888	-4.609818	0.44959	-1.018024
28	H	4.507138	0.848292	-0.66057	-4.492966	-1.129714	-1.797785
29	N	-3.28536	-2.07448	0.018807	3.280591	-1.89826	0.382725
30	O	-0.00135	-0.78055	-0.31312	-0.057694	-0.841289	-0.164848
31	O	0.070773	2.838527	-0.27422	-0.31803	2.740025	-0.617699

32	O	-4.69632	-0.23348	0.022322	4.539383	0.039903	0.576471
33	O	-2.69561	2.328801	-0.03576	2.449356	2.431528	-0.149126
34	O	-1.3183	-3.22779	0.790364	1.628451	-3.232632	-0.749948

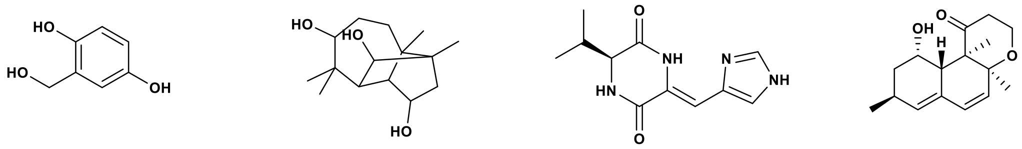

S1(Gentisyl alcohol) S2(5-hydroxyculmorin) **S3**(Pre-aurantiamine) **S4**(Versiol)

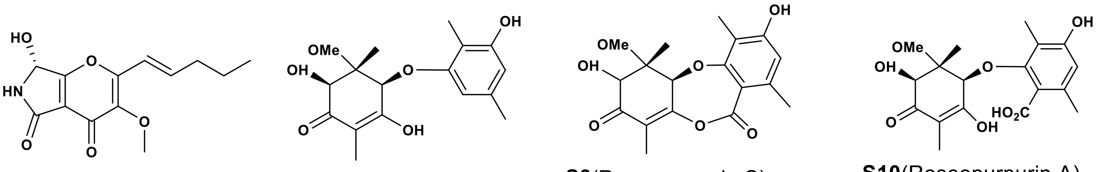

S5(Pyranonigrin G) **S6**(Aculeatusquinone C) **S8**(Roseopurpurin C) **S10**(Roseopurpurin A)

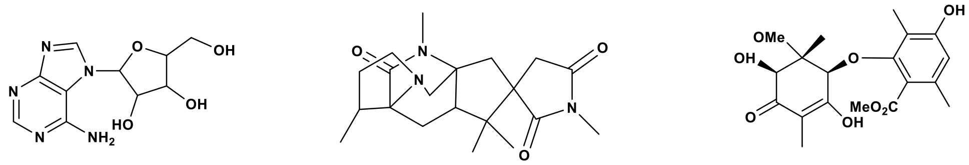

S7(Adenosine) S9(Aspergillimide) **S11**(Roseopurpurin B)

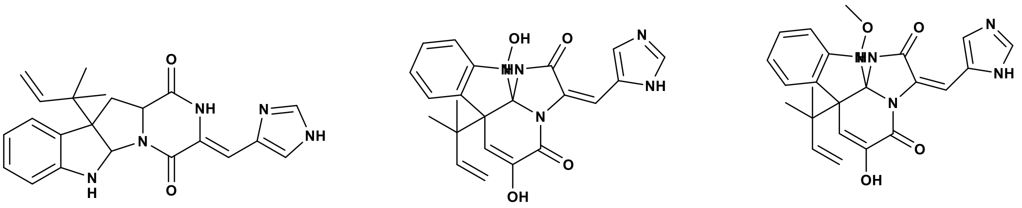

S12(Roquefortine C) S13(Glandicoline B) S14(Meleagrine)

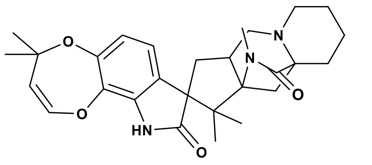

S15(Marcfortine A)

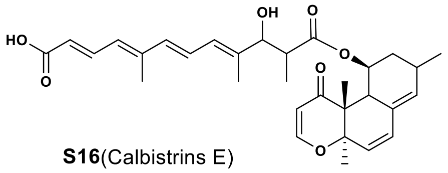

S16(Calbistrins E)

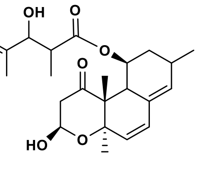

S17(Calbistrins A)

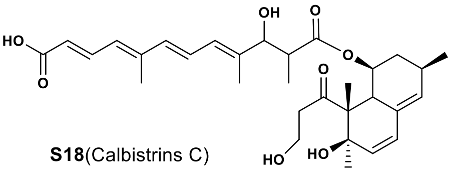

S18(Calbistrins C)

Fig.S13 Total of 18 features induced only after the CER were identified by the combination of the computational approach.

Compounds information

pyranoginrin G **(S5)**: brown oil; HRESIMS m/z 266.1016 $[M+H]^+$ (calcd for $C_{13}H_{15}O_5N$, 265.1016); ^{1}H and ^{13}C NMR as shown in Table 2 and S4-S5;

roseopurpurin A **(S10)**: white powder; HRESI(+)MS m/z 367.1376 $[M+H]^+$, HRESI(-)MS m/z 365.1235 $[M-H]^-$ (calcd for $C_{18}H_{22}O_8$, 366.1315).^{1}H-NMR (500 MHz, DMSO-*d6*):1.24 (3H, s), 1.57(3H, s), 1.96 (3H, s), 2.14 (3H, s), 3.05 (3H, s), 4.27(1H, s), 4.64(1H, s), 6.39(1H, s), 9.52(1H, s), 10.26(1H, s), 12.33(1H, s). ^{13}C-NMR (125.76 MHz, DMSO-*d6*):7.61, 9.56, 13.79, 19.59, 50.02, 70.18, 80.43, 83.31, 107.09, 110.95, 114.20, 119.44, 132.91, 154.78, 156.43, 169.11, 171.96, 192.7.

roseopurpurin B **(S11)**: brown powder; HRESI(+)MS m/z 381.1536 $[M+H]^+$, HRESI(-)MS m/z 379.1395 $[M-H]^-$ (calcd for $C_{19}H_{24}O_8$, 380.1417).^{1}H-NMR (500 MHz, DMSO-*d6*):1.16 (3H, s), 1.57(3H, s), 1.99 (3H, s), 2.10 (3H, s), 3.00 (3H, s), 3.68 (3H, s), 4.34(1H, s), 4.60(1H, s), 6.41(1H, s), 9.61(1H, s), 10.85(1H, s). ^{13}C-NMR (125.76 MHz, DMSO-*d6*):7.66, 9.44, 13.45, 19.50, 49.90, 51.51, 71.12, 80.94, 83.40, 106.89, 111.33, 114.21, 118.14, 133.45, 155.51, 157.04, 168.19,171.94.

roseopurpurin C **(S8)**: white powder; HRESI(+)MS m/z 349.1273 $[M+H]^+$, HRESI(-)MS m/z 347.1130 $[M-H]^-$ (calcd for $C_{18}H_{20}O_7$, 348.1209).^{1}H-NMR (500 MHz, DMSO-*d6*):1.01(3H, s), 1.77(3H, d, *J* =1.7Hz), 2.08 (3H, s), 2.38(3H, s), 3.28 (3H, s), 4.32(1H, s), 5.35(1H, d, *J* =1.7Hz), 5.53(1H, d, *J* =3.9Hz), 6.62(1H, s), 10.62(1H, s). ^{13}C-NMR (125.76 MHz, DMSO-*d6*):8.31, 8.41, 13.31, 21.82, 50.13, 74.65, 80.28, 82.75, 110.88, 113.67, 114.53, 117.12, 142.04, 159.15, 160.12, 160.70, 161.11, 197.47.

aculeatusquinone C **(S6)**: white powder; HRESI(+)MS m/z 323.1480 $[M+H]^+$, HRESI(-)MS m/z 321.1342 $[M-H]^-$ (calcd for $C_{17}H_{22}O_6$, 322.1416).^{1}H-NMR (500 MHz, DMSO-*d6*):1.10 (3H, s), 1.61(3H, s), 1.98 (3H, s), 2.12 (3H, s), 3.13 (3H, s), 4.64(1H, s), 5.07(1H, s), 6.19(1H, s), 6.24(1H, s), 8.99(1H, s), 10.09(1H, s). ^{13}C-NMR (125.76 MHz, DMSO-*d6*):7.75, 8.39, 12.89, 21.23, 50.12, 81.20, 81.89, 105.02, 106.97, 108.49, 109.31, 134.66, 155.35, 157.96, 171.93,189.90.

Pre-aurantiamine **(S3)**: brown oil; HRESI(+)MS m/z 235.1177 $[M+H]^+$, HRESI(-)MS m/z 233.1039 $[M-H]^-$ (calcd for $C_{11}H_{14}O_2N_4$, 234.1117). ^{1}H-NMR (500 MHz, DMSO-*d6*):0.83 (3H, d, *J*=6.8Hz), 0.93 (3H, d, *J*=7.1Hz), 2.16 (1H, m), 3.94 (1H, t, *J*=2.8Hz), 6.52(1H, s), 7.46 (1H, s), 7.92 (1H, s), 8.27 (1H, s), 11.56 (1H, s), 12.55 (1H, s). ^{13}C-NMR (125.76 MHz, DMSO-*d6*):16.55, 18.04, 33.28, 60.16, 103.31, 118.53, 124.64, 136.35 136.47, 159.31, 164.76.

Versiol **(S4)**: white powder; HRESI(+)MS m/z 263.1637 $[M+H]^+$(calcd for $C_{16}H_{22}O_3$, 262.1569).^{1}H-NMR (500 MHz, DMSO-*d6*):0.96(3H, s), 0.97(3H, d, *J*=7.3Hz), 1.10(3H, s), 1.24(1H, m), 1.75(1H, dt, *J*=12.9, 4.4Hz), 2.09(1H, dd, *J*=14.2, 3.0), 2.52(1H, m), 3.00(1H, ddd, *J*=14.2, 12.3, 8.7Hz), 3.29(1H, q, *J*=2.9Hz), 3.78(2H, m), 3.96(1H, dd, *J*=11.5, 8.7Hz), 4.21(1H, d, *J*=3.0Hz), 5.37(1H, d, *J*=9.6Hz), 5.62(1H, s), 6.12(1H, d, *J*=9.6Hz).^{13}C-NMR (125.76 MHz, DMSO-*d6*):12.88, 20.0, 20.93, 25.25, 38.57, 39.91, 40.81, 56.5, 59.97, 64.99, 78.59, 128.55, 130.86, 131.33, 134.1, 210.23.

Calbistrin A **(S17)** : light yellow powder; HRESI(+)MS m/z 563.2590 $[M+Na]^+$ (calcd for $C_{31}H_{40}O_8$, 540.2723);^{1}H-NMR (500 MHz, MeOD): 0.96 (3H, d, *J*=7.1 Hz), 1.09 (3H, d, *J*=7.1 Hz), 1.28 (3H, s), 1.33 (1H, m),1.38 (3H, s), 1.81 (3H, s), 2.08 (3H, s), 2.24 (1H, m), 2.44 (1H, d, *J*=3.9), 2.47 (1H, d, *J*=3.9), 2.59 (1H, m), 2.87 (1H, dd, *J*=14.0,8.6Hz), 2.93(1H,m), 4.14 (1H,d,*J*=9.8Hz),5.27(1H,dd, *J*=8.6,3.9Hz), 5.69(1H, d, *J*=9.8Hz), 5.79(1H, m), 5.94(1H, d, *J*=15.0Hz), 6.05(2H, m), 6.17(1H, d, *J*=11.4 Hz), 6.33(1H, d, *J*=11.9Hz), 6.44(1H, d, *J*=15.2Hz), 6.77(1H, dd, *J*=15.2,11.4Hz), 7.72(1H, dd, *J*=15.0,11.9Hz). ^{13}C-NMR (125.76 MHz, MeOD): 11.68, 13.04, 14.78, 18.38, 21.29, 23.40,

28.18, 36.39, 41.20, 45.87, 54.99, 71.26, 75.40, 80.91, 92.52, 122.31, 128.40, 129.03, 129.55, 129.86, 131.63, 131.72, 136.70, 137.95, 141.18, 141.74, 145.30, 171.08,176.68, 211.19

Calbistrin C **(S18)**: light yellow powder; HRESI(+)MS m/z 563.2590 $[M+Na]^+$ (calcd for $C_{31}H_{42}O_8$, 542.2880);^{1}H-NMR (500 MHz, MeOD): 0.91 (3H, d, *J*=7.1 Hz), 1.03 (3H, d, *J*=7.1 Hz), 1.14 (3H, s), 1.26 (1H, m),1.41 (3H, s), 1.79 (3H, s), 2.05 (3H, s),2.09 (1H, m), 2.45 (1H, m), 2.56 (1H, m), 2.80 (1H, dt, *J*=18.0,5.9Hz), 3.03 (1H, dt, *J*=18.0,6.7Hz), 3.15 (1H, m), 3.76(1H, dt, *J*=10.9,5.9Hz), 3.82(1H, dt, *J*=10.9,6.7Hz) 4.10 (1H, d, *J*=9.8 Hz), 5.39(2H, m), 5.65(1H, s), 5.89(1H, d, *J*=15.0 Hz), 5.97(1H, d, *J*=9.8 Hz), 6.15(1H, d, *J*=11.0 Hz), 6.30(1H, d, *J*=11.9 Hz), 6.42(1H, d, *J*=15.2 Hz), 6.75(1H, dd, *J*=15.2,11.0 Hz), 7.70(1H, dd, *J*=15.0,11.9 Hz).^{13}C-NMR (125.76 MHz, MeOD): 11.59, 13.05, 14.42, 14.80, 21.42, 26.51, 27.54, 36.75, 42.21, 45.11, 45.83, 58.38, 58.63, 71.07, 75.34, 81.04, 122.27, 128.42, 129.01, 129.65, 129.81, 132.44, 134.55, 134.85, 137.94, 141.19, 141.77, 145.30, 171.11,176.97, 215.67.
